# IRSp53 Deletion in Glutamatergic and GABAergic Neurons and in Male and Female Mice Leads to Distinct Electrophysiological and Behavioral Phenotypes

**DOI:** 10.3389/fncel.2020.00023

**Published:** 2020-02-11

**Authors:** Yangsik Kim, Young Woo Noh, Kyungdeok Kim, Esther Yang, Hyun Kim, Eunjoon Kim

**Affiliations:** ^1^Graduate School of Medical Science and Engineering, Korea Advanced Institute of Science and Technology (KAIST), Daejeon, South Korea; ^2^Department of Biological Sciences, Korea Advanced Institute of Science and Technology (KAIST), Daejeon, South Korea; ^3^Department of Anatomy, College of Medicine, Korea University, Seoul, South Korea; ^4^Center for Synaptic Brain Dysfunctions, Institute for Basic Science (IBS), Daejeon, South Korea

**Keywords:** autism, synapse, IRSp53, mPFC, social interaction, hyperactivity

## Abstract

IRSp53 (also known as BAIAP2) is an abundant excitatory postsynaptic scaffolding protein implicated in autism spectrum disorders (ASD), schizophrenia, and attention-deficit/hyperactivity disorder (ADHD). IRSp53 is expressed in different cell types across different brain regions, although it remains unclear how IRSp53 deletion in different cell types affects brain functions and behaviors in mice. Here, we deleted IRSp53 in excitatory and inhibitory neurons in mice and compared resulting phenotypes in males and females. IRSp53 deletion in excitatory neurons driven by *Emx1* leads to strong social deficits and hyperactivity without affecting anxiety-like behavior, whereas IRSp53 deletion in inhibitory neurons driven by *Viaat* has minimal impacts on these behaviors in male mice. In female mice, excitatory neuronal IRSp53 deletion induces hyperactivity but moderate social deficits. Excitatory neuronal IRSp53 deletion in male mice induces an increased ratio of evoked excitatory and inhibitory synaptic transmission (E/I ratio) in layer V pyramidal neurons in the prelimbic region of the medial prefrontal cortex (mPFC), whereas the same mutation does not alter the E/I ratio in female neurons. These results suggest that IRSp53 deletion in excitatory and inhibitory neurons and in male and female mice has distinct impacts on behaviors and synaptic transmission.

## Introduction

IRSp53 (encoded by *Baiap2*) is a multi-domain scaffolding or adaptor protein that is abundantly present in the postsynaptic density of excitatory synapses (Sheng and Kim, 2011; Kang et al., 2016). IRSp53 directly interacts with PSD-95 and Shank, excitatory postsynaptic scaffolding proteins known to regulate synapse assembly and function and implicated in various brain disorders, including autism spectrum disorders (ASD; Sheng and Sala, 2001; Sheng and Hoogenraad, 2007; Jiang and Ehlers, 2013; Sala et al., 2015; Monteiro and Feng, 2017). Functionally, IRSp53 regulates dendritic spines and synaptic function through its ability to coordinate Rac and Cdc42 small GTPase-dependent modulation of actin filaments (Kang et al., 2016), the main cytoskeleton of dendritic spines (Sala and Segal, 2014).

Global deletion of IRSp53 in mice decreases dendritic spine density in the cortex and induces abnormal behaviors, including social-interaction deficits, hyperactivity, and cognitive impairments (Sawallisch et al., 2009; Chung et al., 2015), in line with the reported implication of IRSp53/BAIAP2 in ASD (Celestino-Soper et al., 2011; Levy et al., 2011; Toma et al., 2011), schizophrenia (Fromer et al., 2014; Purcell et al., 2014), and attention-deficit/hyperactivity disorder (ADHD; Ribasés et al., 2009; Liu et al., 2013). In addition, global IRSp53 deletion in mice abnormally increases the function of N-methyl-D-aspartate receptors (NMDARs) in the hippocampus, and the NMDAR antagonist memantine improves social deficits in IRSp53-mutant mice (Kim et al., 2009; Chung et al., 2015; Bobsin and Kreienkamp, 2016), supporting the growing importance of NMDAR dysfunction in ASD (Lee et al., 2015). A previous study has shown that IRSp53 is expressed in various cell types, including excitatory neurons in the neocortex and GABAergic neurons in the striatum and cerebellum (Burette et al., 2014). However, the impacts of cell type-specific IRSp53 expression on brain functions and behaviors, including social interaction, remain essentially unknown.

Here, we restricted *Irsp53* knockout (KO) in dorsal telencephalic glutamatergic neurons using *Emx1*-Cre mice and GABAergic neurons using *Viaat*-Cre mice, and found that glutamatergic *Irsp53* KO led to social deficits and hyperactivity associated with increased ratio of evoked excitatory and inhibitory synaptic transmission (E/I ratio) in the medial prefrontal cortex (mPFC) of male mice. In female mice, glutamatergic *Irsp53* KO led to moderate social deficits that are associated with an unaltered cortical E/I ratio.

## Materials and Methods

### Animals

Mice were bred and maintained according to the Requirements of Animal Research at KAIST. All procedures were approved and followed by the Committee of Animal Research at KAIST (KA201). We used male mice for behavioral, electrophysiological, and other (biochemical, FISH, and tdTomato expression in Emx1- and Viaat-Cre mice) experiments; female mice were also used for behavioral tests [three-chamber and open-field test (OFT)] and electrophysiology.

Mice were fed *ad libitum*, and 2–4 mice were housed together in a cage under a 12-h light-dark cycle. There were no differences in the body weights of age-matched mouse groups. Mice were identified by polymerase chain reaction (PCR) genotyping using the following PCR primers: IRSp53 flox AGGAGGTGTTTCTGCTCTGG/AATAGCAGTCTGGGGTCTGG; Cre CGTACTGACGGTGGGAGAAT/TGCATGATCTCCGGTATTGA.

### Behavioral Assays

All behavioral assays were performed using age-matched C57BL6/J mice (8–16 weeks) generated by Cre/+; *Irsp53*^flox/+^ × *Irsp53*^flox/flox^ mating. All behavioral assays were performed during light-off periods. The light condition for all behavioral assays was ~120 lux. There were at least 1 day-long rest periods between tests. The behavioral assays were performed in the order of the open field test, elevated plus-maze (EPM) test, and three-chamber social interaction test. Behavioral assays were recorded as video files (.avi format) and analyzed by Ethovision XT 10 (Noldus, The Netherlands).

### Three-Chamber Social Interaction Test

The three-chambered social-interaction test was performed as described previously (Moy et al., 2004; Silverman et al., 2010). The apparatus had the following dimensions; W 60 × H 40 × D 20 cm for the whole apparatus, and W 20 × H 20 × D 20 cm for each chamber. The side chambers contained an aluminum grid with a curved face to confine the mouse/object. The assay consisted of three sessions. During the first 10-min session, a subject mouse was allowed to freely explore all three chambers for habituation. Then the mouse was confined briefly in the center chamber, while a novel object and a WT stranger mouse, stranger 1, were placed in the side chambers behind the aluminum grid in a random manner to minimize the influences of side bias. The subject mouse was then allowed to freely explore all three chambers for 10 min. Before the last session, the subject mouse was again gently guided to the center chamber while the object was replaced with another WT mouse, stranger 2. The subject mouse was again allowed to freely explore all three chambers for 10 min.

In a modified three-chamber social interaction test performed for five consecutive days to measure social novelty in mice (Bariselli et al., 2018), we used the same apparatus and social interaction scheme. This test used an empty aluminum grid without an object, unlike the conventional three-chamber social interaction test. A subject mouse was exposed to the first stranger for the first 4 days to maximize habituation to the stranger, and the stranger was placed in alternate chambers to suppress the effect of side bias. One day 5, the first stranger was replaced with the second stranger to measure social-novelty recognition.

All stranger mice were age-matched males and were habituated to the side chambers in advance during the previous day for 30 min. The positions of the object and stranger mouse were alternated between tests to minimize the influences of side preference.

### Open-Field Test

Mice were placed in the center region of an open-field box (40 × 40 × 40 cm). Open-field locomotor activities were measured for 60 min.

### Elevated Plus-Maze Test

An elevated-plus maze was made of gray acryl with four arms, each 30-cm long and 5-cm wide (Walf and Frye, 2007). The height of the maze was elevated 75 cm above the ground. The light condition of closed arms was ~0 lux. A test mouse was placed in the center of the maze at the junction of the four arms in the beginning and was allowed to freely explore the maze for 10 min.

### Whole-Cell Recordings

Coronal slices (mPFC) were prepared using a vibratome (VT1200S, Leica, Germany) in ice-cold dissection buffer (in mM: 212 sucrose, 25 NaHCO_3_, 5 KCl, 1.25 NaH_2_PO_4_, 10 D-glucose, 2 sodium pyruvate, 1.2 sodium ascorbate, 3.5 MgCl_2_, 0.5 CaCl_2_ bubbled with 95% O2/5% CO_2_). The slices were recovered at 32 °C in normal artificial cerebrospinal fluid (ACSF; in mM: 125 NaCl, 25 NaHCO_3_, 2.5 KCl, 1.25 NaH_2_PO_4_, 10 D-glucose, 1.3 MgCl_2_, 2.5 CaCl_2_) and thereafter maintain at room temperature. Cells were visualized using infrared differential interference contrast video microscopy (Olympus, BX50XI). Whole-cell current-clamp recordings were made by using a MultiClamp 700B amplifier (Molecular Devices).

For voltage-clamp recordings, recording pipettes (3–5 MΩ) were filled with a solution containing (in mM) 120 CsMeSO_4_, 15 CsCl, 10 TEA-Cl, 8 NaCl, 10 HEPES, 0.25 EGTA, 5 QX-314, 4 MgATP, and 0.3 NaGTP, pH 7.25–7.35 (280–300 mOsm; Rothwell et al., 2014). Signals were filtered at 2 kHz and digitized at 10 kHz. Miniature excitatory postsynaptic currents (mEPSCs) were recorded in the presence of AP5 (50 μM) and tetrodotoxin (1 μM) at the holding potential of −70 mV. Miniature inhibitory postsynaptic currents (mIPSCs) were recorded at the holding potential of 0 mV, as described previously (Liang et al., 2015). For voltage-clamp recordings with electrical stimulation (NMDA/AMPA ratio, paired-pulse ratio, and excitatory/inhibitory ratio), a stimulus pipette was located 100 μm toward the pia from the patched cell. Stimulus electrode was soaked in the abovementioned bath solution, and 1/15 Hz stimulation was used to obtain baseline responses (20/8/20 for NMDA/AMPA ratio, paired-pulse ratio, and excitatory/inhibitory ratio, respectively). Stimulus intensity was modified in different experiments (NMDA/AMPA ratio, <10 pA at 50 ms after stimulation; paired-pulse ratio, 60 pA < EPSC1 < 200 pA).

For current-clamp recordings, recording pipettes (3–5 MΩ) were filled with a solution containing (in mM) 120 Kgluconate, 20 HEPES, 0.4 EGTA, 2.8 NaCl, 5 TEA-Cl, 2.5 MgATP, and 0.25 NaGTP, pH 7.25–7.35 (280–300 mOsm). Picrotoxin (100 μM) and NBQX (10 μM) were present throughout the experiments to block inhibitory and excitatory synaptic transmissions, respectively. If the series resistance changed by more than 20%, data were not included in the analysis. Membrane potentials were not corrected for junction potentials (estimated to be 10 mV). To obtain sustained firings, a series of current (1 s duration, 50 pA steps for mPFC) was injected. To measure action potential thresholds, a series of current steps (2 ms duration at 2.5 Hz, 0–2,500 pA range, +10 pA step increments) were injected into patched neurons until an action potential was generated. To measure the input resistance, hyperpolarizing current steps (1 s duration, 0 to −100 pA, −25 pA step increments) were injected into patched neurons. All voltage measures were taken after neurons had reached a stable response (Chen et al., 2013).

### Immunoblotting and Immunofluorescence

For immunoblotting experiments, a fresh brain was homogenized with ice-cold lysis buffer containing 320 mM sucrose, 10 mM HEPES pH 7.4, 5 mM EDTA, and protease inhibitors. For immunofluorescence experiments, isoflurane-anesthetized mice were transcardially perfused with 4% paraformaldehyde in phosphate-buffered saline (PFA/PBS), followed by brain removal and incubation in 4% PFA/PBS for 24 h for fixation. Fixed brains were sectioned (100 μm) using a vibratome (VT1200S, Leica, Germany) and subjected to immunofluorescence staining for IRSp53. The following antibodies were purchased commercially: BAIAP2/IRSp53 antibody (1:1,000, Atlas, rabbit, HPA023310), and α-tubulin antibody (1:10,000, Sigma, mouse, T9026). For immunoblotting, secondary antibodies for BAIAP2/IRSp53 and β-tubulin antibodies were donkey anti-rabbit antibody with 800 nm detection (LiCor, 1:10,000) and donkey anti-mouse antibody with HRP (Jackson, 1:10,000), respectively.

### Fluorescent *in situ* Hybridization

Frozen sections (14 μm thick) were cut coronally through the hippocampal formation. The sections were thaw-mounted onto Superfrost Plus Microscope Slides (Thermo Fisher Scientific, Waltham, MA, USA; 12-550-15). The sections were fixed in 4% formaldehyde for 10 min, dehydrated in increasing concentrations of ethanol for 5 min, and finally air-dried. Tissues were then pretreated for protease digestion for 10 min at room temperature. For RNA detection, incubations with different amplifier solutions were performed in a HybEZ hybridization oven (ACDBio, Newark, CA, USA) at 40°C. The probes used in this study were three synthetic oligonucleotides complementary to the nucleotide (nt) sequence 2–1,268 of Mm-Baiap2-C1, nt 464–1,415 of Mm-Slc17a7/Vglut1-C2, nt 1986–2,998 of Mm-Slc17a6/Vglut2-C3, nt 62–3,113 of Mm-Gad1-C3, nt 552–1,506 of Mm-Gad2-C2 (ACDBio, Newark, CA, USA). The labeled probes were conjugated to Atto 550 (C1), Alexa Fluor 488 (C2), and Atto 647 (C3). The sections were hybridized at 40°C with labeled probe mixtures (C1 + C2 + C3) per slide for 2 h. Then the non-specifically hybridized probes were removed by washing the sections, three times each in 1× wash buffer at room temperature for 2 min. Amplification steps involved sequential incubations with Amplifier 1-FL for 30 min, Amplifier 2-FL for 15 min, Amplifier 3-FL for 30 min, and Amplifier 4 Alt B-FL at 40°C for 15 min. Each amplifier solution was removed by washing three times with 1× wash buffer for 2 min at room temperature. Fluorescent images were acquired using TCS SP8 Dichroic/CS (Leica), and the ImageJ program (NIH) was used to analyze the images.

### Statistics

Statistical data analysis was performed using Prism 6 (GraphPad). Data normality was determined using the Shapiro-Wilk normality test. Data with normal distribution were analyzed using Student’s *t*-test and analysis of variance (ANOVA), followed by *post hoc* tests. Data failing the normality test were analyzed using the Mann–Whitney test. ROUT method was used to exclude outliers with a Q coefficient of 1%. Exact numbers of mice used and the statistical details are shown in Supplementary Table S1.

## Results

### *Irsp53* mRNA Expression in Glutamatergic and GABAergic Neurons in the Cortex

**Figure 1:**
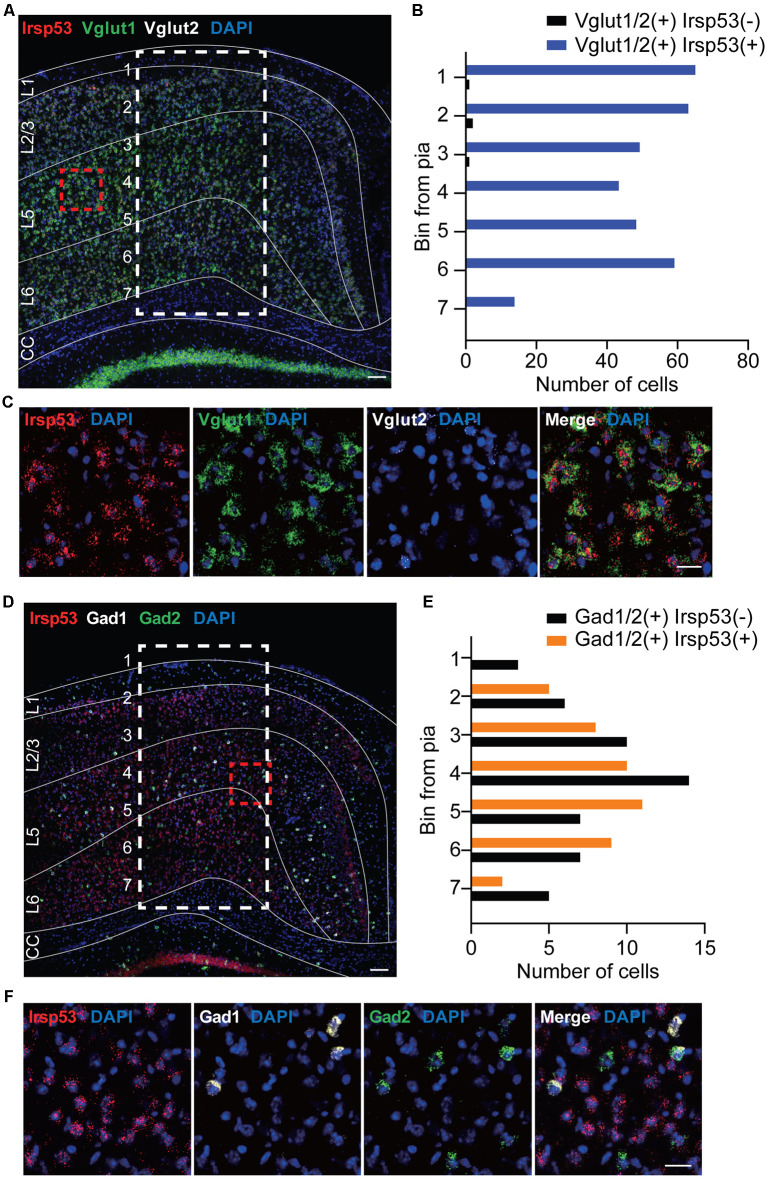
*Irsp53* mRNAs are primarily detected in glutamatergic but minimally in GABAergic neurons in the cortex. **(A–F)** Coronal sections of WT mice (8 weeks) were subjected to fluorescence *in situ* hybridization. Note that *Irsp53*/*Baiap2* mRNAs are detected in *Vglut1/2*-positive glutamatergic neurons but minimally in *Gad1/2*-positive GABAergic neurons in the motor and somatosensory cortex. DAPI was used for nuclear staining. Red dashed line boxes were enlarged to show the levels of neuronal colabelings, and white dashed line boxes with subdivisions across cortical depth were used to quantify the colabelings. L1, cortical layer 1; CC, corpus callosum. Scale bar, 100 μm **(A,D)** and 25 μm **(C,F)**.

To explore specific brain cell types that contribute to social deficits and hyperactivity observed in global *Irsp53*-KO mice (Chung et al., 2015), we first determined IRSp53 expression in glutamatergic and GABAergic neurons by *in situ* fluorescence hybridization. *Irsp53* mRNA was readily detected in *Vglut1/2*-positive glutamatergic neurons in the cortex, but it was minimally detectable in *Gad1/2*-positive GABAergic neurons (Figures 1A–F). Quantitative analysis indicated that colabelings of *Irsp53* and *Vglut1/2* mRNAs were not different across the depth of cortical layers, whereas colabelings of *Irsp53* and *Gad1/2* mRNAs, although sparse (~13% of the *Irsp53*-*Vglut1/2* colabelings in number), were stronger in middle layers. These results are consistent with the reported expression of IRSp53 protein primarily in glutamatergic but not GABAergic neurons in the cortex and hippocampus, although IRSp53 protein is also detectable in striatal and cerebellar GABAergic neurons (Burette et al., 2014).

### *Irsp53* Deletion in Dorsal Telencephalic Glutamatergic but Not GABAergic Neurons Induces Social Deficits and Hyperactivity

**Figure 2:**
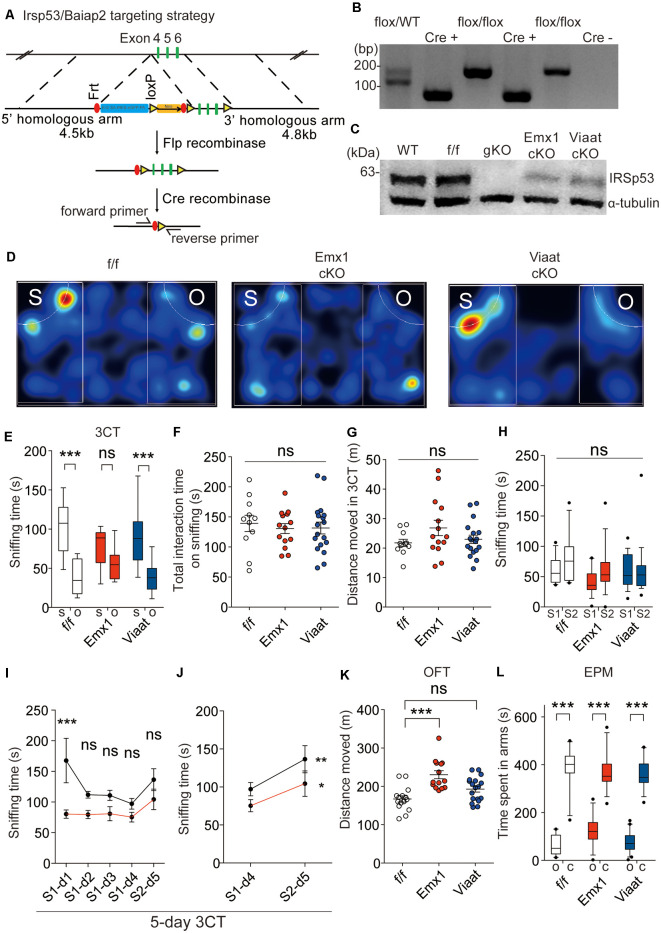
*Irsp53* deletion in glutamatergic but not GABAergic neurons induces social deficits and hyperactivity. **(A)** Schematic for conditional *Irsp53/Baiap2* knockout (KO). **(B)** Polymerase chain reaction (PCR) genotyping of *Irsp53*^fl/fl^ mice and conditional *Irsp53*-KO mice (*Emx1-Cre;Irsp53*^fl/fl^ or *Viaat-Cre;Irsp53*^fl/fl^ mice) using primer sets directed for the Irsp53/Baiap2 allele and Cre recombinase. The two bands in the WT/flox lane represent PCR products from *Irsp53* alleles with and without the Frt + LoxP sites. **(C)** Levels of IRSp53 proteins in global IRSp53-KO (gKO) mice, *Emx1-Cre;Irsp53*^fl/fl^ mice (Emx1 cKO), and *Viaat-Cre;Irsp53*^fl/fl^ mice (Viaat cKO). Whole-brain lysates from mice at P56 were used for immunoblotting. **(D–G)** Suppressed social interaction in *Emx1-Cre;Irsp53*^fl/fl^ but not *Viaat-Cre;Irsp53*^fl/fl^ mice (2 months; males) in the three-chamber social interaction test (3CT), as shown by time spent sniffing social/object target, as compared with control *Irsp53*^fl/fl^ mice. S, social target (stranger mouse); O, object target. Note that total time spent sniffing and distance moved in the 3CT apparatus are normal in the mutant mice. *n* = 11 mice (*Irsp53*^fl/fl^ or f/f), 14 mice (Emx1-cKO), and 18 mice (Viaat-cKO), ***P < 0.001; ns, not significant, two-way analysis of variance (ANOVA) with Bonferroni’s test. 3CT, interaction factor/*F*_(2,94)_ = 5.317, chamber *F*_(1,94)_ = 67.54, genotype *F*_(2,94)_ = 0.7795; total time spent sniffing, *F*_(2,40)_ = 0.1732; total distance moved, *F*_(2,40)_ = 1.824. **(H)** Lack of social novelty recognition in *Emx1-Cre;Irsp53*^fl/fl^, *Viaat-Cre;Irsp53*^fl/fl^, and control (*Irsp53*^fl/fl^) mice (2 months; males) in the three-chamber test. S1, old stranger; S2, new stranger. *n* = 11 mice (*Irsp53*^fl/fl^ or f/f), 14 mice (Emx1-cKO), and 18 mice (Viaat-cKO), ns, not significant, two-way ANOVA with Bonferroni’s test, interaction *F*_(2,80)_ = 0.8983, chamber *F*_(1,80)_ = 3.370, genotype *F*_(2,80)_ = 1.632. **(I,J)** Normal social novelty recognition in *Emx1-Cre;Irsp53*^fl/fl^ mice (2 months; males) in the 5-day three-chamber test, as shown by the difference in sniffing time for an old stranger (S1) and a new stranger (S2) on day 4 and 5, respectively. Note that control (*Irsp53*^fl/fl^) mice show normal levels of habituation to S1, as shown by the time sniffing S1 across days 1–4 that becomes insignificant on days 2–4. *n* = 8 mice (*Irsp53*^fl/fl^ or f/f), eight mice (Emx1-cKO), *P < 0.05, **P < 0.01, ***P < 0.001; ns, not significant, two-way ANOVA with Bonferroni’s test and repeated-measures AVOVA with Bonferroni’s test. Five-day three-chamber test, day 1–5, interaction *F*_(4,28)_ = 1.769, time *F*_(4,28)_ = 3.103, genotype *F*_(1,7)_ = 29.86; day 4–5, interaction *F*_(1,7)_ = 0.9518, time *F*_(1,7)_ = 8.614, genotype *F*_(1,7)_ = 11.62. **(K)** Increased locomotor activity in *Emx1-Cre;Irsp53*^fl/fl^ but not in *Viaat-Cre;Irsp53*^fl/fl^ mice (2 months; males) in the open-field test (OFT). *n* = 15 mice (f/f), 14 mice (Emx1-cKO), and 18 mice (Viaat-cKO), ***P < 0.001; ns, not significant, one-way ANOVA with Bonferroni’s test, *F*_(2,44)_ = 12.60. **(L)** Normal anxiety-like behavior in *Emx1-Cre;Irsp53*^fl/fl^ and *Viaat-Cre;Irsp53*^fl/fl^ mice (2 months; males) in the elevated plus-maze (EPM) test, as shown by time spent in open/closed arms. O, open arm; C, closed arm. *n* = 15 mice (f/f), 14 mice (Emx1-cKO), and 18 mice (Viaat-cKO), ***P < 0.001, two-way ANOVA with Bonferroni’s test, interaction *F*_(2,88)_ = 3.848, arm *F*_(1,88)_ = 485.2, genotype *F*_(2,88)_ = 1.518.

For conditional *Irsp53* KO in glutamatergic or GABAergic neurons, we generated a novel mouse line in which exons 4–6 of *Irsp53* are floxed (*Irsp53*^fl/fl^ mice) and crossed them with *Emx1-Cre* (Jax005628; dorsal telencephalic glutamatergic; Gorski et al., 2002) and *Viaat-Cre* (Jax017535; Chao et al., 2010) mice, respectively (Figure 2A). The resulting conditional *Irsp53*-KO mouse lines, *Emx1-Cre; Irsp53*^fl/fl^ and *Viaat-Cre; Irsp53*^fl/fl^, were verified by PCR genotyping and immunoblot analysis (Figures 2B,C). IRSp53 protein levels in *Emx1-Cre; Irsp53*^fl/fl^ and *Viaat-Cre; Irsp53*^fl/fl^ whole brains were ~29 ± 4% and ~64 ± 4% of WT values, respectively. Appropriate expression of Cre in the mouse lines used in this study was confirmed by crossing with a reporter mouse line (Ai9 tdTomato line; JAX 007909; Madisen et al., 2010; Supplementary Figure S1).

In behavioral experiments performed using male mice, *Emx1-Cre;Irsp53^fl/fl^* mice displayed impaired social interaction in the three-chamber test compared with control (*Irsp53*^fl/fl^) mice without Cre expression (Figures 2D,E). These changes did not accompany altered total social interaction or locomotor activity in the three-chamber apparatus (Figures 2F,G).

Changes in social novelty recognition during the three-chamber test could not be determined because control *Irsp53^fl/fl^* mice did not prefer to explore a novel stranger (Figure 2H). However, an additional test for social novelty recognition termed 5-day three-chamber test, where a subject mouse was exposed to the first stranger mouse for four consecutive days for full habituation followed by exposure to the second stranger mouse on day 5 (Bariselli et al., 2018), *Emx1-Cre;Irsp53*^fl/fl^ mice displayed normal social novelty recognition that is comparable to that of control (*Irsp53*^fl/fl^) mice (Figures 2I,J).

*Emx1-Cre;Irsp53^fl/fl^* mice displayed hyperactivity in the OFT but normal anxiety-like behavior in the EPM test (Figures 2K,L). *Viaat-Cre;Irsp53*^fl/fl^ mice showed no detectable changes in social interaction, locomotor activity, or anxiety-like behavior (Figures 2D,E,L). Control (*Irsp53*^fl/fl^) mice showed normal social interaction and locomotor activity, compared with WT mice (without *Irsp53*^fl/fl^ and Cre alleles; Supplementary Figures 2A,B). In addition, mice expressing Cre alone (*Emx1-Cre* and *Viaat-Cre*) showed normal social interaction, locomotion, or anxiety-like behavior (Supplementary Figures 2C–E). Therefore, *Irsp53* KO in dorsal telencephalic glutamatergic, but not GABAergic, neurons leads to social deficits and hyperactivity in mice, similar to those in global *Irsp53*-KO mice (Chung et al., 2015).

### *Emx1-Cre; Irsp53^fl/fl^* and *Viaat-Cre; Irsp53*^fl/fl^ Mice Show Distinct Changes in Synaptic Transmission and Intrinsic Excitability in mPFC Pyramidal Neurons

**Figure 3:**
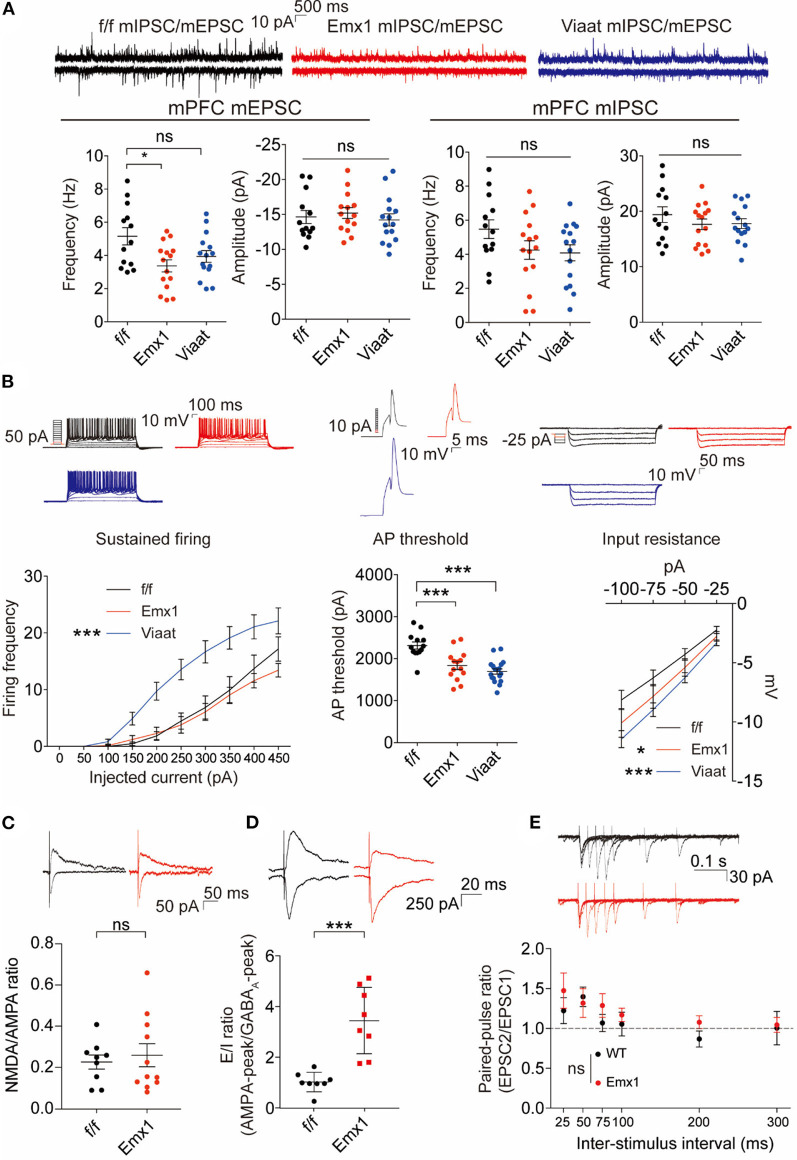
*Emx1-Cre;Irsp53*^fl/fl^ and *Viaat-Cre;Irsp53*^fl/fl^ mice show distinct changes in synaptic transmission and intrinsic excitability in medial prefrontal cortex (mPFC) pyramidal neurons. **(A)** Miniature excitatory postsynaptic currents (mEPSCs) and miniature inhibitory postsynaptic currents (mIPSCs) in layer V pyramidal neurons in the prelimbic region of the mPFC in *Emx1-Cre;Irsp53*^fl/fl^ and *Viaat-Cre;Irsp53*^fl/fl^ mice (3 months; male). Note that the frequency of mEPSCs is significantly decreased in *Emx1-Cre;Irsp53*^fl/fl^ mice. *n* = 13 neurons from three mice for f/f-mEPSC, 14, 3 for Emx1-mEPSC, 15, 3 for Viaat-mEPSC, 13, 3 for f/f-mIPSC, 15, 3 for Emx1-mIPSC, and 15, 3 for Viaat-mIPSC, *P < 0.05, ns, not significant, one-way ANOVA with Bonferroni’s test. mEPSC frequency, *F*_(2,39)_ = 4.119; mEPSC amplitude, *F*_(2,39)_ = 0.342; mIPSC frequency, *F*_(2,40)_ = 2.012; mIPSC amplitude, *F*_(2,40)_ = 0.7806. **(B)** Intrinsic excitability in layer V pyramidal neurons in the prelimbic region of the mPFC in *Emx1-Cre;Irsp53*^fl/fl^ and *Viaat-Cre;Irsp53*^fl/fl^ mice (3 weeks; male). Note that intrinsic excitability is increased both in *Emx1-Cre;Irsp53*^fl/fl^ and *Viaat-Cre;Irsp53*^fl/fl^ mice. *n* = 13, 3 for f/f-firing frequency, 14, 3 for Emx1-firing frequency, 18, 3 for Viaat-firing frequency, 13, 3 for f/f-AP threshold, 14, 3 for Emx1-AP threshold, 18, 3 for Viaat-AP threshold, 13, 3 for f/f-input resistance, 14,3 for Emx1-input resistance, 18, 3 for Viaat-input resistance, *P < 0.05, ***P < 0.001; ns, not significant, one-way ANOVA with Bonferroni’s test for AP threshold, two-way ANOVA with Bonferroni’s test for firing frequency and input resistance. Sustained firing, interaction *F*_(18,420)_ = 3.165, current *F*_(9,420)_ = 61.89, genotype *F*_(2,420)_ = 56.73; action potential threshold, *F*_(2,42)_ = 16.14; input resistance, interaction *F*_(6,168)_ = 0.5088, current *F*_(3,168)_ = 60.88, genotype *F*_(2,168)_ = 11.33. **(C)** Normal ratio of evoked N-methyl-D-aspartate receptors (NMDAR)-EPSCs and AMPA receptor (AMPAR)-EPSCs in *Emx1-Cre;Irsp53*^fl/fl^ layer V pyramidal neurons in the prelimbic region of the mPFC (2 months; male). *n* = 9 neurons for three mice for f/f, 11, 3 for Emx1, ns, not significant, Student’s t-test, t = 0.2447, df = 18. **(D)** Increased ratio of evoked EPSCs and IPSCs in *Emx1-Cre;Irsp53*^fl/fl^ layer V pyramidal neurons in the prelimbic region of the mPFC (2 months; male). *n* = 8 neurons for three mice for f/f, 8, 3 for Emx1, ***P < 0.001, Student’s *t*-test, *t* = 5.019, *df* = 14. **(E)** Normal paired-pulse ratio in *Emx1-Cre;Irsp53*^fl/fl^ layer V pyramidal neurons in the prelimbic region of the mPFC (2 months; male). *n* = 10 neurons for three mice for f/f, 9, 3 for Emx1, ns, not significant, two-way ANOVA with Bonferroni’s test, interaction *F*_(5,85)_ = 0.6379, time *F*_(5,85)_ = 4.100, genotype *F*_(1,17)_ = 0.7348.

To explore mechanisms underlying the social deficits and hyperactivity in *Emx1-Cre; Irsp53^fl/fl^* mice, we analyzed synaptic and neuronal properties in the mPFC, a brain region that displayed decreased excitatory synapse density in global *Irsp53*-KO mice (Chung et al., 2015). The frequency but not amplitude of mEPSCs in the *Emx1-Cre; Irsp53*^fl/fl^ mPFC (layer V pyramidal neurons in the prelimbic area) was decreased, whereas mIPSCs were normal (Figure 3A), in line with the reported decrease in excitatory synaptic transmission and dendritic spine density in mPFC pyramidal neurons from *Irsp53*-null mice (Chung et al., 2015). In addition, these neurons showed moderately increased intrinsic excitability, as shown by action potential threshold and input resistance (Figure 3B), likely to compensate for the decreased excitatory synaptic input. In *Viaat-Cre; Irsp53*^fl/fl^ mice, however, mEPSCs or mIPSCs were normal in layer V mPFC neurons (Figure 3A). Intriguingly, the intrinsic excitability was strongly increased, as shown by current-firing curve, action potential threshold, and input resistance (Figure 3B).

When evoked synaptic transmission was measured, the ratio of NMDAR-mediated EPSCs and AMPA receptor (AMPAR)-mediated EPSCs was not altered in *Emx1-Cre; Irsp53^fl/fl^* layer V pyramidal neurons (Figure 3C). These results collectively suggest that *Irsp53* deletion in glutamatergic neurons leads to reduced spontaneous excitatory but not inhibitory synaptic transmission, increased ratio of evoked EPSCs/IPSCs, and increased neuronal excitability without affecting evoked NMDAR-EPSC/AMPAREPSC ratio in layer V mPFC neurons.

### Male and Female *Emx1-Cre; Irsp53^fl/fl^* Mice Show Distinct Changes in Synaptic Transmission and Behaviors

**Figure 4:**
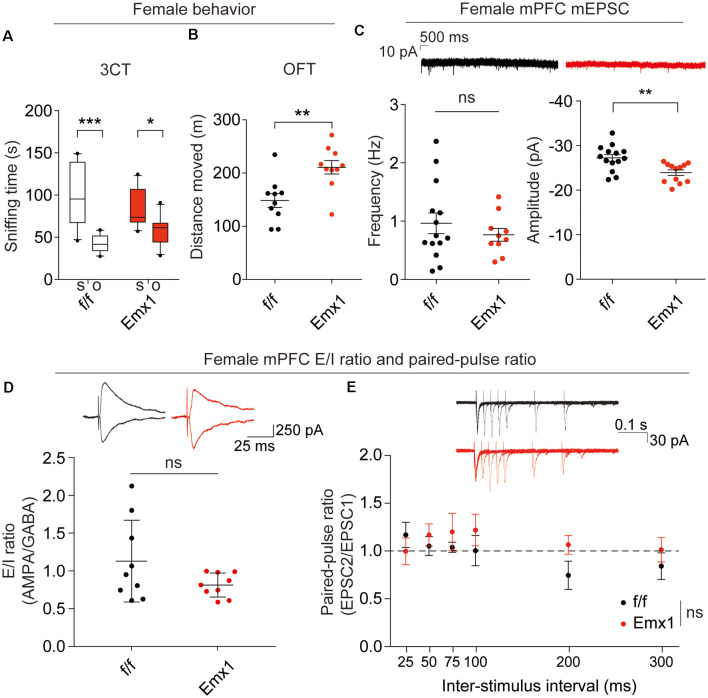
Female *Emx1-Cre;Irsp53^fl/fl^* mice show distinct changes in synaptic transmission and behaviors. **(A)** Normal social interaction in female *Emx1-Cre;Irsp53*^fl/fl^ mice in the three-chamber test (2 months; females). *n* = 10 mice (*Irsp53*^fl/fl^ or f/f), 10 mice (Emx1-cKO), *P < 0.05, ***P < 0.001, two-way ANOVA with Bonferroni’s test, interaction *F*_(1,36)_ = 4.035, chamber *F*_(1,36)_ = 31.58, genotype *F*_(1,36)_ = 0.0003937. **(B)** Hyperactivity in female *Emx1-Cre;Irsp53*^fl/fl^ mice in the OFT (2 months; females). *n* = 10 mice (*Irsp53*^fl/fl^ or f/f), 10 mice (Emx1-cKO), **P < 0.01, Student’s *t*-test, *t* = 3.434, *df* = 18. **(C)** Decreased amplitude but normal frequency of mEPSCs in layer V pyramidal neurons in the prelimbic region of the mPFC in *Emx1-Cre;Irsp53*^fl/fl^ (3 months; females). *n* = 14 neurons from three mice for f/f-mEPSC, 10, 3 for Emx1-mEPSC in mPFC, **P < 0.01; ns, not significant, Mann–Whitney test for frequency, Student’s *t*-test for amplitude. mEPSC frequency, Mann-U 62; mEPSC amplitude, *t* = 3.278, *df* = 24. **(D)** Normal ratio of evoked EPSCs and IPSCs in layer V pyramidal neurons in the prelimbic region of the mPFC in female *Emx1-Cre;Irsp53*^fl/fl^ mice (2–3 months; females). *n* = 9 neurons for three mice for f/f, 9, 3 for Emx1, ns, not significant, Student’s *t*-test, *t* = 1.675, *df* = 16. **(E)** Normal paired-pulse ratio in layer V pyramidal neurons in the prelimbic region of the mPFC in female *Emx1-Cre;Irsp53*^fl/fl^ (2–3 months; females). *n* = 6 neurons for three mice for f/f, 6, 3 for Emx1, ns, not significant, two-way ANOVA with Bonferroni’s test, interaction *F*_(5,90)_ = 0.8666, time *F*_(5,90)_ = 0.9748, genotype *F*_(1,18)_ = 0.6927.

The abovementioned behavioral and electrophysiological results were obtained from male *Emx1-Cre; Irsp53^fl/fl^* mice. Given that male-female differences could affect these phenotypes, we measured social interaction and locomotor activity in *Emx1-Cre;Irsp53*^fl/fl^ mice. Intriguingly, female *Emx1-Cre;Irsp53*^fl/fl^ mice showed normal three-chamber social interaction in the three-chamber test, although there was a decreasing tendency, as compared with control (*Irsp53*^fl/fl^) mice (Figure 4A), indicative of male-female difference in social interaction. In contrast, female *Emx1-Cre;Irsp53*^fl/fl^ mice showed strong hyperactivity in the OFT (Figure 4B), similar to male *Emx1-Cre;Irsp53*^fl/fl^ mice.

When excitatory synaptic transmission was measured in layer V pyramidal neurons in the prelimbic area of the mPFC from female *Emx1-Cre;Irsp53^fl/fl^* mice, there was a decrease in the amplitude, but not frequency, of mEPSCs in female mutant neurons, compared with WT neurons (Figure 4C), which contrasts with the decreased frequency but not amplitude of mEPSCs in male mutant neurons (Figure 3A). In addition, there were no genotype differences in the ratio of evoked EPSCs/IPSCs or the paired-pulse ratio in layer V pyramidal neurons (Figures 4D,E). These results collectively suggest that *Irsp53* deletion induces distinct changes in behaviors and excitatory synaptic transmission in the mPFC.

## Discussion

We attempted here to restrict *Irsp53* deletion to Emx1-positive glutamatergic and Viaat-positive GABAergic neurons to investigate the impact of IRSp53 KO in the respective neurons on mouse behaviors and synaptic/neuronal properties. *Irsp53* KO in Emx1-positive dorsal telencephalic glutamatergic neurons leads to both social interaction deficits and hyperactivity, two key behavioral phenotypes observed in global *Irsp53*-KO mice (Chung et al., 2015), whereas *Irsp53* KO in Viaat-positive GABAergic neurons does not affect social interaction or hyperactivity. Therefore, *Irsp53* expression in glutamatergic neurons in the cortex, where Emx1 is strongly expressed, seems to be important for normal social interaction and locomotor activity. This is in line with the well-known importance of the PFC in the regulation of social cognition and interaction, previously reported in studies with human subjects as well as WT and mutant mice carrying ASD- and schizophrenia-related gene mutations (Ernst et al., 1997; Mundy, 2003; Pierce et al., 2004; Carper and Courchesne, 2005; Amodio and Frith, 2006; Gilbert et al., 2008; Rinaldi et al., 2008; Shalom, 2009; Courchesne et al., 2011; Yizhar et al., 2011; Testa-Silva et al., 2012; Liang et al., 2015; Barak and Feng, 2016; Ko, 2017; Selimbeyoglu et al., 2017; Cao et al., 2018; Pirone et al., 2018; Wang et al., 2018, 2019; Guo et al., 2019; Lazaro et al., 2019; Phillips et al., 2019; Yoo et al., 2019).

*Irsp53* KO restricted to Emx1-positive glutamate neurons induces decreased mEPSC frequency, decreased NMDA/AMPA ratio, and increased E/I ratio in layer V pyramidal neurons in the prelimbic region of the mPFC. These changes are associated with moderately increased neuronal excitability. Whether these changes alter the output function of the mutant layer V pyramidal neurons under basal or social conditions would require additional analyses. However, the mPFC is known to receive afferent projections from various brain regions (Riga et al., 2014; Root et al., 2015; Murugan et al., 2017; Park and Moghaddam, 2017; Knowland and Lim, 2018). In particular, the prelimbic region of the mPFC receives strong afferent projections from limbic regions of the cortex as well as other subcortical areas, including basal forebrain, thalamus, amygdala, hypothalamus, and midbrain (Hoover and Vertes, 2007). In addition, layer V pyramidal neurons in the prelimbic area project to various subcortical regions, including lateral hypothalamus, striatum, and basolateral amygdala (Sesack et al., 1989; Gabbott et al., 2005). Therefore, the altered spontaneous and evoked synaptic transmission and intrinsic excitability of the mutant layer V pyramidal neurons might change their output functions and contribute to social deficits and hyperactivity observed in *Emx1-Cre;Irsp53*^fl/fl^ mice.

*Emx1-Cre;Irsp53^fl/fl^* males show strong social deficits whereas females show only modestly suppressed social interaction, while they both show comparable hyperactivity. This suggests that the hyperactivity is not the key confounding factor contributing to the social deficits. Notably, spontaneous excitatory synaptic transmission is distinctly changed in layer V pyramidal neurons in the prelimbic area in male and female *Emx1-Cre;Irsp53*^fl/fl^ mice; decreased mEPSC frequency in male neurons and decreased mEPSC amplitude (not frequency) in female neurons. This difference, although intriguing, is less likely to induce a qualitative difference in the output function of these neurons. Importantly, however, the E/I ratio of evoked synaptic transmission was increased in male, but female, layer V pyramidal neurons. Although further details remain to be determined, these results are in line with the reported association of altered E/I ratio in cortical neurons with social deficits (Yizhar et al., 2011; Nelson and Valakh, 2015; Lee et al., 2017; Selimbeyoglu et al., 2017). In addition, these results add to the emerging notion that non-sex-differential factors such as synaptic transmission and neuronal properties (relative to sex-differential factors such as hormone and X-Y chromosomes) may contribute to the male-female phenotypic differences in animal models of autism (Werling and Geschwind, 2013; Barak and Feng, 2016; Lo et al., 2016; Werling et al., 2016; Jung et al., 2018). Last, our data indicate that GABAergic neuronal deletion of IRSp53 in male mice minimally affects social and locomotor activities. However, this does not exclude the possibility that female mice with the same mutation show some positive electrophysiological and behavioral phenotypes.

In conclusion, our data suggest that *Irsp53* KOs restricted to glutamatergic neurons and GABAergic neurons and in male and female mice lead to distinct behavioral deficits and changes in synaptic and neuronal properties in the mPFC.

## Data Availability Statement

All datasets generated for this study are included in the article/Supplementary Material.

## Ethics Statement

The animal study was reviewed and approved by the Committee of Animal Research at Korea Advanced Institute of Science and Technology (KAIST).

## Author Contributions

YK performed most of the experiments except for FISH experiments. YK, YN, and KK performed the electrophysiological experiments. EY performed the FISH experiments. YK, HK, and EK designed the experiments and wrote the manuscript.

## Conflict of Interest

The authors declare that the research was conducted in the absence of any commercial or financial relationships that could be construed as a potential conflict of interest.
