# Corrigendum: IRSp53 Deletion in Glutamatergic and GABAergic Neurons and in Male and Female Mice Leads to Distinct Electrophysiological and Behavioral Phenotypes

**DOI:** 10.3389/fncel.2021.782716

**Published:** 2021-11-22

**Authors:** Yangsik Kim, Young Woo Noh, Kyungdeok Kim, Esther Yang, Hyun Kim, Eunjoon Kim

**Affiliations:** ^1^Graduate School of Medical Science and Engineering, Korea Advanced Institute of Science and Technology (KAIST), Daejeon, South Korea; ^2^Department of Biological Sciences, Korea Advanced Institute of Science and Technology (KAIST), Daejeon, South Korea; ^3^Department of Anatomy, College of Medicine, Korea University, Seoul, South Korea; ^4^Center for Synaptic Brain Dysfunctions, Institute for Basic Science (IBS), Daejeon, South Korea

**Keywords:** autism, synapse, IRSp53, mPFC, social interaction, hyperactivity

In the original article, there was a mistake in Figure 3 as published. It was due to an inadvertent mistake in the quantification process. The new quantification indicates that there is no statistical difference in the NMDA/AMPA ratio between WT and IRSp53-KO mice; previous Figure 3C indicated a decrease in the mutant mice. The correct Figure 3 and legend appears below.

**Figure 3:**
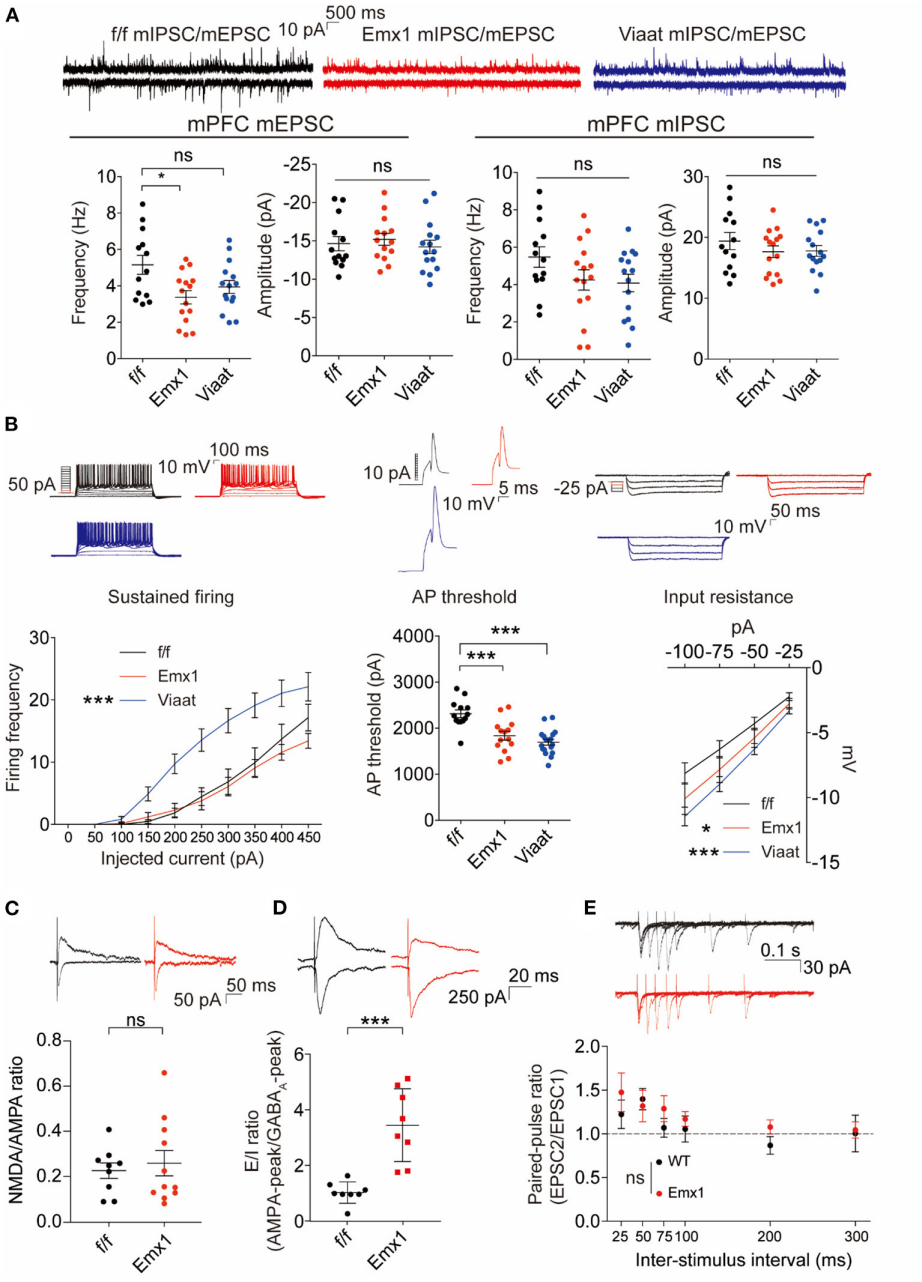
*Emx1-Cre;Irsp53*^*fl*/*fl*^ and *Viaat-Cre;Irsp53*^*fl*/*fl*^ mice show distinct changes in synaptic transmission and intrinsic excitability in medial prefrontal cortex (mPFC) pyramidal neurons. **(A)** Miniature excitatory postsynaptic currents (mEPSCs) and miniature inhibitory postsynaptic currents (mIPSCs) in layer V pyramidal neurons in the prelimbic region of the mPFC in *Emx1-Cre;Irsp53*^*fl*/*fl*^ and *Viaat-Cre;Irsp53*^*fl*/*fl*^ mice (3 months; male). Note that the frequency of mEPSCs is significantly decreased in *Emx1-Cre;Irsp53*^*fl*/*fl*^ mice. *n* = 13 neurons from three mice for f/f-mEPSC, 14, 3 for Emx1-mEPSC, 15, 3 for Viaat-mEPSC, 13, 3 for f/f-mIPSC, 15, 3 for Emx1-mIPSC, and 15, 3 for Viaat-mIPSC, *P < 0.05, ns, not significant, one-way ANOVA with Bonferroni's test. mEPSC frequency, *F*_(2, 39)_ = 4.119; mEPSC amplitude, *F*_(2, 39)_ = 0.342; mIPSC frequency, *F*_(2, 40)_ = 2.012; mIPSC amplitude, *F*_(2, 40)_ = 0.7806. **(B)** Intrinsic excitability in layer V pyramidal neurons in the prelimbic region of the mPFC in *Emx1-Cre;Irsp53*^*fl*/*fl*^ and *Viaat-Cre;Irsp53*^*fl*/*fl*^ mice (3 weeks; male). Note that intrinsic excitability is increased both in *Emx1-Cre;Irsp53*^*fl*/*fl*^ and *Viaat-Cre;Irsp53*^*fl*/*fl*^ mice. *n* = 13, 3 for f/f-firing frequency, 14, 3 for Emx1-firing frequency, 18, 3 for Viaat-firing frequency, 13, 3 for f/f-AP threshold, 14, 3 for Emx1-AP threshold, 18, 3 for Viaat-AP threshold, 13, 3 for f/f-input resistance, 14,3 for Emx1-input resistance, 18, 3 for Viaat-input resistance, *P < 0.05, ***P < 0.001; ns, not significant, one-way ANOVA with Bonferroni's test for AP threshold, two-way ANOVA with Bonferroni's test for firing frequency and input resistance. Sustained firing, interaction *F*_(18, 420)_ = 3.165, current *F*_(9, 420)_ = 61.89, genotype *F*_(2, 420)_ = 56.73; action potential threshold, *F*_(2, 42)_ = 16.14; input resistance, interaction *F*_(6, 168)_ = 0.5088, current *F*_(3, 168)_ = 60.88, genotype *F*_(2, 168)_ = 11.33. **(C)** Normal ratio of evoked N-methyl-D-aspartate receptors (NMDAR)-EPSCs and AMPA receptor (AMPAR)-EPSCs in *Emx1-Cre;Irsp53*^*fl*/*fl*^ layer V pyramidal neurons in the prelimbic region of the mPFC (2 months; male). *n* = 9 neurons for three mice for f/f, 11, 3 for Emx1, ns, not significant, Student's *t*-test, *t* = 0.2447, df = 18. **(D)** Increased ratio of evoked EPSCs and IPSCs in *Emx1-Cre;Irsp53*^*fl*/*fl*^ layer V pyramidal neurons in the prelimbic region of the mPFC (2 months; male). *n* = 8 neurons for three mice for f/f, 8, 3 for Emx1, ***P < 0.001, Student's *t*-test, *t* = 5.019, df = 14. **(E)** Normal paired-pulse ratio in *Emx1-Cre;Irsp53*^*fl*/*fl*^ layer V pyramidal neurons in the prelimbic region of the mPFC (2 months; male). *n* = 10 neurons for three mice for f/f, 9, 3 for Emx1, ns, not significant, two-way ANOVA with Bonferroni's test, interaction *F*_(5, 85)_ = 0.6379, time *F*_(5, 85)_ = 4.100, genotype *F*_(1, 17)_ = 0.7348.

To reflect this change a correction has also been made to the Results, *Emx1-Cre; Irsp53*^*fl*/*fl*^ and *Viaat-Cre; Irsp53*^fl/fl^ Mice Show Distinct Changes in Synaptic Transmission and Intrinsic Excitability in mPFC Pyramidal Neurons, Second paragraph:

“When evoked synaptic transmission was measured, the ratio of NMDAR-mediated EPSCs and AMPA receptor (AMPAR)-mediated EPSCs was not altered in *Emx1-Cre; Irsp53*^*fl*/*fl*^ layer V pyramidal neurons (Figure 3C). These results collectively suggest that Irsp53 deletion in glutamatergic neurons leads to reduced spontaneous excitatory but not inhibitory synaptic transmission, increased ratio of evoked EPSCs/IPSCs, and increased neuronal excitability without affecting evoked NMDAR-EPSC/AMPAR-EPSC ratio in layer V mPFC neurons.”

The authors apologize for this error and state that this does not change the scientific conclusions of the article in any way. The original article has been updated.

## Publisher's Note

All claims expressed in this article are solely those of the authors and do not necessarily represent those of their affiliated organizations, or those of the publisher, the editors and the reviewers. Any product that may be evaluated in this article, or claim that may be made by its manufacturer, is not guaranteed or endorsed by the publisher.

