# Supplementary material for: IRSp53 Deletion in Glutamatergic and GABAergic Neurons and in Male and Female Mice Leads to Distinct Electrophysiological and Behavioral Phenotypes
Source: Front Cell Neurosci. 2020 Feb 11;14:23. doi: 10.3389/fncel.2020.00023 (PMC7026675; doi:10.3389/fncel.2020.00023)
Supplement: Supplementary file 2 [file Image_1.pdf]

IRSp53 deletion in glutamatergic and GABAergic neurons and in male and female mice leads to distinct electrophysiological and behavioral phenotypes

Yangsik Kim[1,*], Young Woo Noh[2,*], Kyungdeok Kim[2], Esther Yang[3], Hyun Kim[3], Eunjoon Kim[2, 4,*]

[1]Graduate School of Medical Science and Engineering, Korea Advanced Institute of Science and Technology (KAIST), Daejeon, South Korea; [2]Department of Biological Sciences, Korea Advanced Institute of Science and Technology, Daejeon, South Korea; [3]Department of Anatomy, College of Medicine, Korea University, Seoul 136-705, Korea; [4]Center for Synaptic Brain Dysfunctions, Institute for Basic Science (IBS), Daejeon, South Korea; *These authors contributed equally to the work.

Keywords: autism, synapse, IRSp53, mPFC, social interaction, hyperactivity

Corresponding author:
Eunjoon Kim, PhD
Director and Professor
Center for Synaptic Brain Dysfunctions, Institute for Basic Science (IBS) and Department of Biological Sciences, Korea Advanced Institute of Science and Technology (KAIST)
Kuseong-dong, Yuseong-ku
Daejeon 34141
South Korea
Tel: +82-42-350-2633
Fax: +82-42-350-8127
E-mail: kime@kaist.ac.kr

Supplementary figure legends

Supplementary Figure 1. Validation of *Emx1-Cre* and *Viaat-Cre* mouse lines by tdTomato expression.

(A–C) *Emx1-Cre* or *Viaat-Cre* lines used in the current study were crossed with a reporter mouse line (Ai 9 tdTomato line, JAX 007909), followed by visualization of tdTomato expression in various brain regions in sagittal (a) and coronal (b and c) sections. Scale bar, 1 mm.

Supplementary Figure 2. Control *Irsp53*$^{fl/fl}$ mice and Cre-alone mice show normal levels of social interaction and locomotion.

(A and B) Control *Irsp53*$^{fl/fl}$ mice (2 months) without Cre expression were subjected to three-chamber social interaction and open-field tests. n = 8 mice for WT and 11 mice for f/f, ***P < 0.001, ns, not significant, two-way ANOVA with Bonferroni's test for three chamber test, Mann-Whitney test for open field test.

(C–E) Control Cre-alone mice (Emx1 and Viaat; 2 months) were subjected to three-chamber social interaction, open-field, and elevated plus-maze tests. n = 8 mice for WT, 8 for Emx1, and 8 for Viaat, *P < 0.05, **P < 0.01, ***P < 0.001, ns, not significant, one-way ANOVA with Bonferroni's test for open-field and two-way ANOVA with Bonferroni's test for three-chamber social interaction and elevated plus-maze tests.

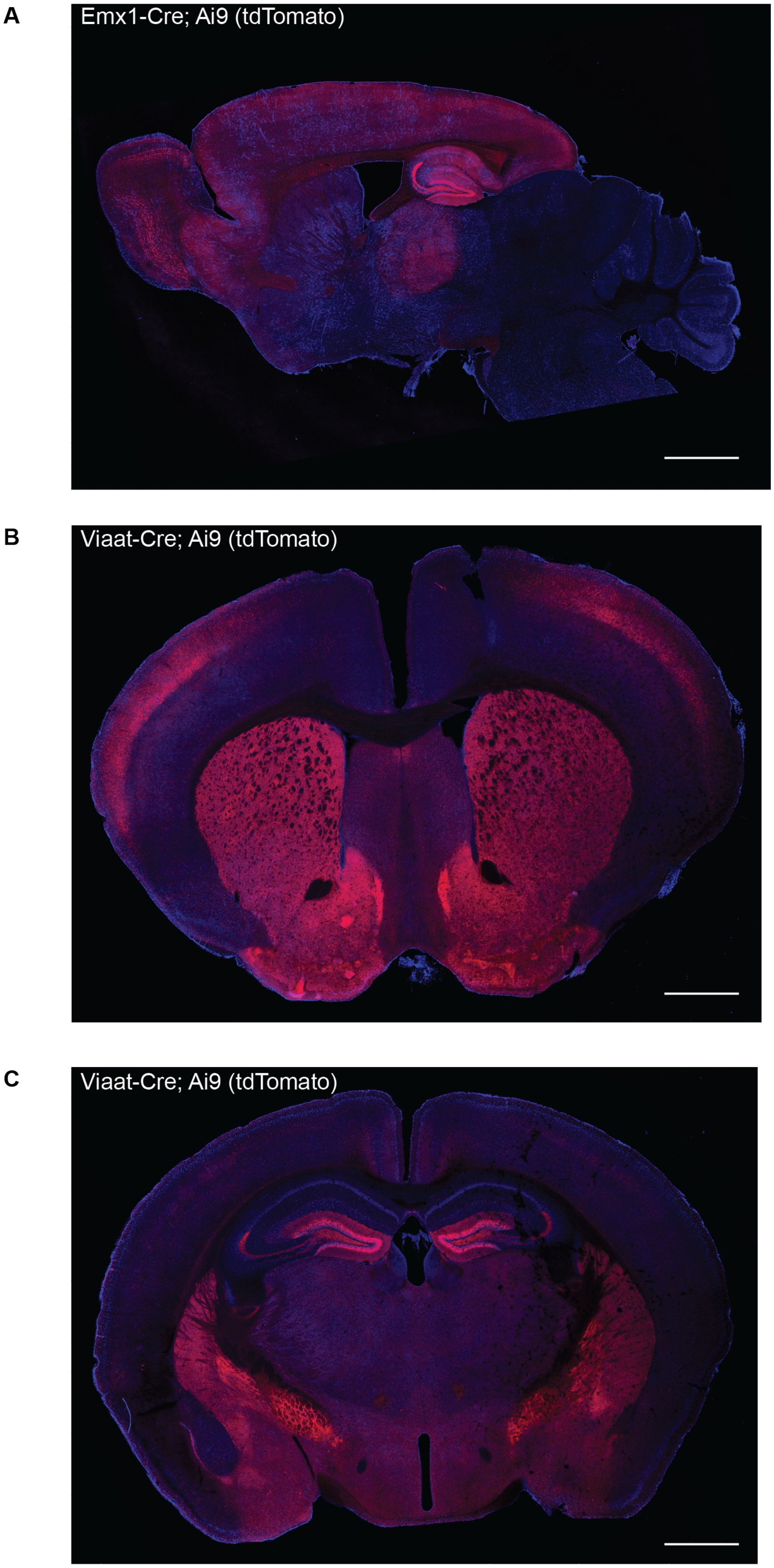
A
Emx1-Cre; Ai9 (tdTomato)
B
Viaat-Cre; Ai9 (tdTomato)
C
Viaat-Cre; Ai9 (tdTomato)

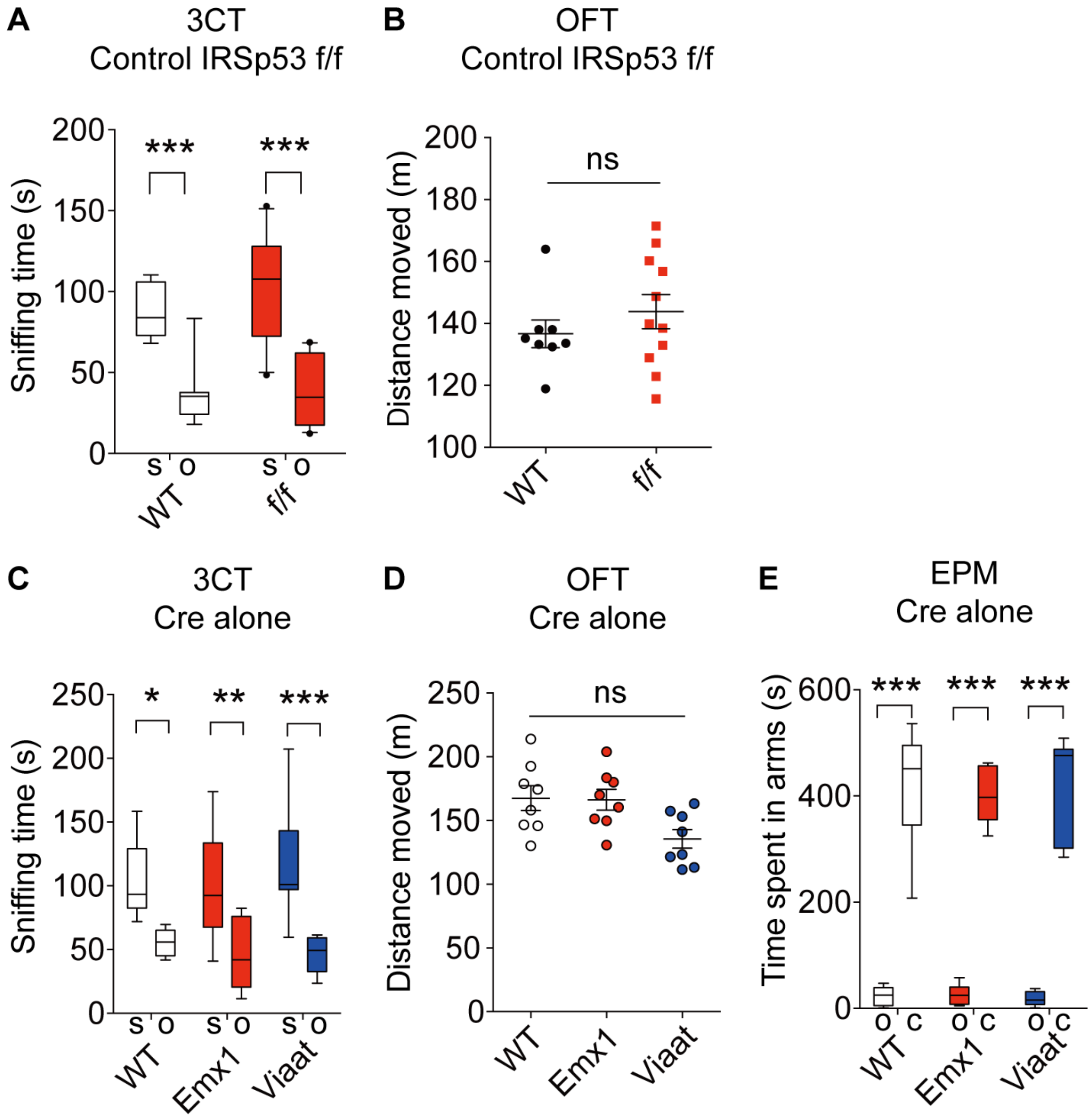
A
3CT
Control IRSp53 f/f
Sniffing time (s)
***
***
s o s o
WT f/f
B
OFT
Control IRSp53 f/f
Distance moved (m)
ns
WT f/f
C
3CT
Cre alone
Sniffing time (s)
*
**
***
s o s o s o
WT Emx1 Viaat
D
OFT
Cre alone
Distance moved (m)
ns
WT Emx1 Viaat
E
EPM
Cre alone
Time spent in arms (s)
***
***
***
o c o c o c
WT Emx1 Viaat
